# Extracellular Vesicle cystatin c is associated with unstable angina in troponin negative patients with acute chest pain

**DOI:** 10.1371/journal.pone.0237036

**Published:** 2020-08-05

**Authors:** Mirthe Dekker, Farahnaz Waissi, Joelle van Bennekom, Max J. M. Silvis, Nathalie Timmerman, Arjan H. Schoneveld, Diederick E. Grobbee, Robbert J. de Winter, Arend Mosterd, Leo Timmers, Dominique P. V. de Kleijn

**Affiliations:** 1 Department of Vascular Surgery, University Medical Centre Utrecht, Utrecht, the Netherlands; 2 Department of Cardiology, Amsterdam University Medical Centre, Amsterdam, the Netherlands; 3 Department of Cardiology, University Medical Centre Utrecht, Utrecht, the Netherlands; 4 Department of Clinical Chemistry and Haematology, University Medical Centre Utrecht, Utrecht, the Netherlands; 5 Julius Center for Health Sciences and Primary Care, University Medical Centre Utrecht, Utrecht, the Netherlands; 6 Department of Cardiology, Meander Medical Centre Amersfoort, Amersfoort, the Netherlands; 7 Department of Cardiology, St. Antonius Hospital Nieuwegein, Nieuwegein, the Netherlands; 8 Netherlands Heart Institute, Utrecht, the Netherlands; Erasmus Medical Center, NETHERLANDS

## Abstract

**Background:**

Despite the use of high-sensitive cardiac troponin there remains a group of high-sensitive cardiac troponin negative patients with unstable angina with a non-neglectable risk for future adverse cardiovascular events, emphasising the need for additional risk stratification. Plasma extracellular vesicles are small bilayer membrane vesicles known for their potential role as biomarker source. Their role in unstable angina remains unexplored. We investigate if extracellular vesicle proteins are associated with unstable angina in patients with chest pain and low high-sensitive cardiac troponin.

**Methods:**

The MINERVA study included patients presenting with acute chest pain but no acute coronary syndrome. We performed an exploratory retrospective case-control analysis among 269 patients. Cases were defined as patients with low high-sensitive cardiac troponin and proven ischemia. Patients without ischemia were selected as controls. Blood samples were fractionated to analyse the EV proteins in three plasma-subfractions: TEX, HDL and LDL. Protein levels were quantified using electrochemiluminescence immunoassay.

**Results:**

Lower levels of (adjusted) EV cystatin c in the TEX subfraction were associated with having unstable angina (OR 0.93 95% CI 0.88–0.99).

**Conclusion:**

In patients with acute chest pain but low high-sensitive cardiac troponin, lower levels of plasma extracellular vesicle cystatin c are associated with having unstable angina. This finding is hypothesis generating only considering the small sample size and needs to be confirmed in larger cohort studies, but still identifies extracellular vesicle proteins as source for additional risk stratification.

## Introduction

An Acute Coronary Syndrome (ACS) remains a major cause of disability and death worldwide [1–3]. ACS comprises three clinical phenotypes: ST-Elevated Myocardial Infarction (STEMI), non-ST-Elevated Myocardial Infarction (NSTEMI) and Unstable Angina (UA). With the use of contemporary high-sensitive cardiac troponin (hsTn) assays, the early and rapid diagnosis of myocardial infarction has improved. As a consequence, the number of patients suffering from UA decreased and the number of NSTEMI patients increased. In 2003, 42% of ACS was due to UA, after implementation of hsTn assays in 2010, this number was decreased to 28% [4]. Recent studies show an incidence of UA of 7–9% [5–8]. In contrast, however, the SWEDEHEART registry showed a relative increase of patients with UA with 13% after the implementation of hsTn [9]. Either way, UA has not (yet) become a rare diagnosis after implementation of newer more sensitive troponin assays.

For a long time NSTEMI and UA were often considered to exist along a continuous spectrum. They are thought to represent the same patient characteristics, underlying pathophysiology and outcome, in therapeutic guidelines they are even considered as one entity [1, 2]. However, recent studies show substantial differences between UA and NSTEMI patients with regards to incidence and mortality. Moreover, the non-existence of myocardial injury in UA patients even suggests differences in pathophysiology [10]. Several studies showed higher rates of future MI and coronary revascularisation in UA patients compared with non UA/non ACS patients [10–12]. These findings emphasise the need for additional risk stratification to identify patients with low hsTn levels but true UA, since they have a non-neglectable risk of future adverse cardiovascular events [7].

One way to improve risk stratification, would be the use of biomarkers. A relative unexplored biomarker source are plasma extracellular vesicles (EVs). EVs are approximately 50-1000nm in size, contain a lipid bilayer membrane, and include exosomes, microvesicles and micro particles [13]. All human cells are able to produce EVs. EVs contain a bioactive content (mRNA, miRNA, proteins and lipid particles) reflecting the cell of origin. Previous studies have shown their role in (patho)physiological processes [14, 15], as well as associations between specific EV plasma proteins and future cardiovascular risk [16, 17]. The role of EV proteins in patients with acute chest pain and low levels of high sensitive cardiac troponin I (hs-cTnI) is unknown. We therefore aimed to investigate whether five selected proteins (serpin C1, CD14, serpin G1, cystatin c and serpin F2) in three different plasma subfractions are associated with UA in patients presenting with acute chest pain and low levels of hsTn.

## Methods

### Study population

**Fig 1:**
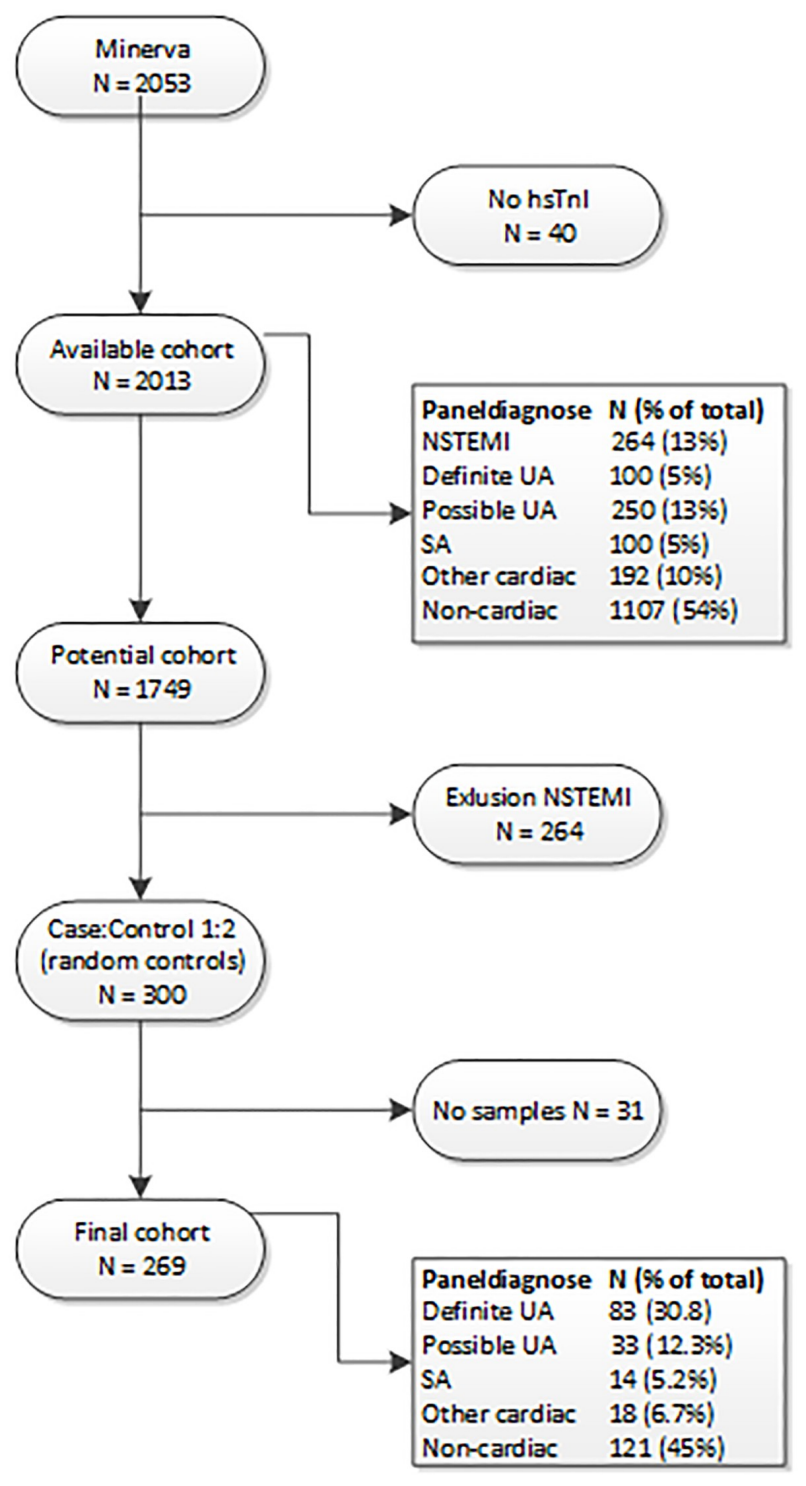
Flowchart study population. *UA* = *Unstable Angina*, *SA* = *Stable angina*.

This study is an exploratory sub analysis of the prospectively collected data from the MINERVA study. Study details are described in detail previously [18]. Shortly, consecutive patients >18 years presenting with acute chest pain at the emergency room (ER) were included. STEMI patients were excluded from participation and additionally, NSTEMI patients were excluded from this sub analysis. Patients were enrolled in the Meander Medical Centre (Amersfoort, the Netherlands) between January 2012 and June 2014. Written informed consent was obtained from all participants. The study has been approved by the Medical Ethics Committee United (MEC-U) and is conform the Declaration of Helsinki. Our primary aim was to compare patients with proven unstable angina (UA) with patients with chest pain but without ACS or proven ischemia (non-UA). The presence of ACS was determined according to the leading ESC guidelines [19]. We performed a nested case-control analysis in which all cases (UA) were selected and twice as much random controls (Fig 1).

### Data collection

Clinical data, e.g. symptoms at presentation, medical history, cardiovascular risk factors, ECG and results from additional testing were all collected and recorded in an online electronic case record form. Clinical decision making was left to the attending cardiologist. Venous blood was collected at presentation at the ER. Blood tubes were centrifuged 10 minutes at 1850xg at room temperature after collection and directly stored at -80°C.

### Adjudication of final diagnosis

Adjudication of the final diagnosis was done by two independent cardiologists from the Meander Medical Centre. A third cardiologist was incorporated in case of disagreement. To adjudicate the final diagnosis all available diagnostic information during the index visit (hs-cTnI, CK-MB, ECG, discharge letters) as well as hospital information (e.g. radiology reports, echocardiography, non-invasive ischemia detection, or coronary angiograms) were considered. Non-invasive ischemia comprised evidence of ischemia on rest ECG, exercise tests, myocardial perfusion imaging or coronary CT. To determine whether or not these tests were positive for ischemia, general guidelines were used. Patients were diagnosed in one of the following five groups: (1) Definite UA, (2) Possible UA, (3) Stable Angina, (4) Other cardiac or (5) non cardiac. Definite UA was defined as patients with typical symptoms: AP at rest; deterioration of previous stable angina and proven ischemia in combination with low hs-cTnI (<60ng/L, since this was the clinical cut-off for MI in the referring hospital). Hs-cTnI was measured with the access AccuTnI+3 Troponin I assay on the UniCel DxI immunoassay System (Beckman Coulter, Brea, CA, Limit of detection was 10ng/L, 99^th^ percentile was 42ng/L coefficient of variation <10%). Possible UA was defined as typical symptoms but without objective evidence of ischemia. Patients with define UA were defined as cases, all the other diagnosis were defined as controls.

### Extracellular vesicle protein analysis

Identification of the selected proteins: serpin C1 (SC1), CD14, serpin G1 (SG1), cystatin c (CC) and serpin F2 (SF2) is based on previously performed proteomics analysis [16, 20]. All proteins were determined and measured in three subfractions. Detailed description of the isolation and quantification procedure can be found in the supplemental materials (S1 Appendix and S1 Fig). In short, for this analysis samples were thawed and EV’s were isolated in three subfractions. A subset of EVs are co-isolated with Low-Density Lipid particles (LDL) while others co-isolate with High-Density Lipid particles (HDL), which allows separation. In addition, one subfraction is analysed without the LDL and HDL fractionation and is referred to as TEX. 15μL of a standard amount of synthetic liposomes, coated with DSG-PEG2000 (Nanocs) and fluorescently labelled with 18:1 liss rhod pe (Merck), was added to each plasma sample to be able to correct for loss of the pellet during isolation. For the sequential isolation of the subfractions Dextran Sulphate (DS) (MP Biomedicals), Manganese (II) Chloride (MnCl2) (Sigma-Aldrich) solutions, Xtractt buffer (1:4) (Cavadis BV) and Nanomag^®^-D plain, 130mm (1:25) (Micromod) or Nano-mag^®^-D PEG-OH (1:25) (Micromod) in case of TEX were used. Coefficient of variation (CV) was determined for each subfraction, e.g. HDL 9.6%, LDL 6.5% and TEX 6.8%. Full characterization was described previous, to get easy access to this data an EV-Track was created (EVTRACK200044). Quantification of the amount of the selected proteins was performed with an electrochemiluminescence immunoassay (Quickplex SQ120, Meso Scale Discovery, MSD) on specific designed 96-wells plates. Protein concentrations were measured in pg/mL.

### Statistical analysis

Continuous data are expressed as mean ± standard deviation or median with interquartile range, according to the distribution of the data. Categorical data are shown as frequencies with corresponding percentages. Differences in continuous data were compared by either independent t-test or Mann-Whitney U. Dichotomous variables were compared by Chi-square or Fisher’s exact test where appropriate. All EV proteins were standardised per synthetic vesicles and were transformed to achieve normal distributions. Log transformation was performed for CD14 HDL and LDL subfraction, CC HDL subfraction, SC1 HDL and TEX subfraction, SF2 HDL and LDL subfraction and SG1 HDL subfraction. Square root transformation was performed for CD14, CC and SF2 in the TEX subfraction, SC1 LDL subfraction and SG1 LDL and TEX subfraction. Uni- and multivariable logistic regression analysis were performed to determine associations between the selected proteins and UA. The multivariable logistic regression analysis was adjusted for sex and the HEART score. The HEART score consists of 5 components: History of complaints, ECG abnormalitaties, Age, Risk factors and Troponin (S1 Table). The HEART score was chosen, since this is a validated clinical prediction tool, specifically designed to stratify patients with acute chest pain in the ER and their risk for future cardiovascular events [21]. Since there are no clinical cut-off values known, we additionally determined an optimal cut-off value for the significant proteins in order to dichotomise all patients as either high or low. For this we first created a logistic regression model with cystatin c TEX in it and obtained the predicted probabilities for each patient. These values were used to plot the sensitivity and specificity of the protein against each other to determine the optimal cut point (S2 Fig). The (adjusted) OR to identify UA patients will be evaluated again with this new variable. All analyses were performed with R Studio (R Software, version 3.5.1).

## Results

All patients were included in the Meander Medical Centre in Amersfoort, The Netherlands, between January 2012 and June 2014. The complete cohort included 2053 patients, in 40 patients no hsTnI was available. The incidence of definite UA was 5% and NSTEMI was present in 13%. In this study all N(STEMI) patients were excluded, we compared patients with definite UA with twice as much, randomly selected, non-UA patients. In 31 patients (17 cases and 14 controls) there was not enough plasma obtained to perform the EV analysis. The final cohort for this analysis was therefore 269 patients (Fig 1).

Baseline characteristics of definite UA patients vs. random non-UA controls are summarised in Table 1. The mean age of patients with definite UA was 64 and 26.5% were women, both were not statistically different from the non-UA control patients (63 and 24.4%). Patients with definite UA more often had a history of coronary artery disease (59.0% vs. 38.7%, p value 0.003), and coronary revascularisation by percutaneous coronary intervention (44.6% vs. 28.5%, p value 0.015). Patients with UA had more often hypertension (61.4% vs. 46.8%, p value 0.036) had a significantly higher HEART score compared to non-UA patients (4.72 vs. 3.89). Among UA patients the use of aspirin and P2Y12-inhibitors was more common compared to patients without definite UA (62.7% vs. 39.2%, p value 0.001, and 26.5% vs. 13.4% p value 0.015, respectively).

**Table 1:** Baseline characteristics.

	Non-UA	UA	P value
n	186	83	
**Demographics**			
Age	63 (12)	64 (11)	0.429
%Women	45 (24.2)	22 (26.5)	0.801
BMI	26.90 (4.10)	26.75 (3.65)	0.776
**Previous history**			
Coronary artery disease	72 (38.7)	49 (59.0)	0.003
Coronary revascularization	62 (33.3)	42 (50.6)	0.011
%CABG	19 (10.2)	10 (12.0)	0.814
%PCI	53 (28.5)	37 (44.6)	0.015
Kidney disease	1 (0.5)	0 (0.0)	1.000
**Risk factors**			
Smoking	48 (25.8)	20 (24.1)	0.884
Hypertension	87 (46.8)	51 (61.4)	0.036
Hypercholesterolemia	61 (32.8)	38 (45.8)	0.057
Diabetes Mellitus	25 (13.4)	15 (18.1)	0.423
Family history of CAD	59 (31.7)	32 (38.6)	0.340
Heart Score	3.89 (1.27)	4.72 (1.11)	<0.001
**Drug therapy**			
Aspirin	73 (39.2)	52 (62.7)	0.001
P2Y12-inhibitors	25 (13.4)	22 (26.5)	0.015
Statin	91 (48.9)	52 (62.7)	0.051
ACE/AT-Inhibitor	81 (43.5)	40 (48.2)	0.566
Β-blocker	76 (40.9)	45 (54.2)	0.057
Calcium Channel Blocker	29 (15.6)	19 (22.9)	0.203

Values are displayed as mean(sd) or frequency(%), UA = Unstable Angina Pectoris, Non UA contains: other cardiac chest pain CABG = coronary artery bypass graft, PCI = Percutaneous Coronary Intervention, CAD = Coronary artery disease. ACE = Angiotensin Converted Enzyme, AT = Angiotensine.

We compared baseline levels of EV proteins among cases and controls. S2 Table shows the results of this comparison. Baseline levels only differed for serpin G1 in the TEX subfraction (7.75 (1.50) vs. 7.39 (1.13), p value 0.049). In addition, we performed an univariate and multivariable logistic regression analysis of which the results can be found in Table 2. After adjustment for sex and the HEART score (a clinical prediction tool including traditional risk factors, see also S2 Table), a significant association between cystatin c in the TEX subfraction and having UA was found (OR 0.93 95% CI 0.88–0.99). We determined the optimal cut-off (S2 Fig) and we dichotomised the levels of cystatin c (TEX subfraction) for each patient in either high or low. After dichotomisation, cystatin c in the TEX subfraction remained a significant predictor of unstable angina, we found an adjusted OR of 0.41 (95%CI: 0.22–0.70).

**Table 2:** Logistic regression analysis for unstable angina.

	Univariate			Multivariable*		
Biomarker	OR	95% CI	P value	OR	95% CI	P value
CD14 HDL	0.94	0.61–1.46	0.787	0.80	0.51–1.28	0.360
CD14 LDL	1.26	0.68–2.32	0.455	1.17	0.60–2.29	0.649
CD14 TEX	0.97	0.90–1.05	0.420	0.95	0.88–1.03	0.234
Cystatin C HDL	0.96	0.82–1.14	0.652	0.85	0.71–1.02	0.085
Cystatin C LDL	0.74	0.42–1.32	0.310	0.63	0.34–1.18	0.15
Cystatin C TEX	0.95	0.90–1.01	0.090	**0.93**	**0.88–0.99**	**0.015**
Serpin C1 HDL	0.99	0.71–1.37	0.935	0.91	0.65–1.28	0.601
Serpin C1 LDL	0.99	0.99–1.01	0.594	0.99	0.99–1.01	0.774
Serpin C1 TEX	0.78	0.44–1.37	0.387	0.93	0.51–1.69	0.799
Serpin F2 HDL	0.43	0.16–1.18	0.101	0.44	0.14–1.32	0.141
Serpin F2 LDL	0.76	0.27–2.14	0.603	0.88	0.28–2.77	0.827
Serpin F2TEX	0.98	0.96–1.01	0.110	0.99	0.96–1.01	0.191
Serpin G1HDL	0.66	0.39–1.12	0.121	0.68	0.39–1.21	0.189
Serpin G1LDL	0.98	0.88–1.11	0.800	0.99	0.88–1.14	0.988
Serpin G1TEX	0.82	0.68–1.01	0.051	0.85	0.69–1.05	0.123

EV protein levels are displayed as mean(sd). Original assay units were pg/mL. Proteins were transformed to achieve a normal distribution and standardized per synthetic vesicle. Log transformation: CD14HDL and LDL, CC HDL and LDL, SC1 HDL and TEX, SF2 HDL and LDL, SG1 HDL. Square root transformation: CD14TEX, CCTEX, SC1LDL, SF2TEX, SG1LDL.and TEX

*Adjusted for age, sex and heart score (containing: History, ECG, Age, Risk factors, Tropnin) at admission.

## Discussion

In this study we aimed to investigate the potential diagnostic role of EV proteins in patients with acute chest pain and UA but low levels of hs-TnI. We performed a retrospective exploratory analysis measuring EV proteins in plasma subfractions in a large cohort of patients presenting with acute chest pain at the ER. This study showed that EV cystatin c in the TEX subfraction is associated with unstable angina independent of clinical factors represented by the HEART score and sex. In the last years hs-troponins have shown excellent diagnostic accuracy to detect patients with myocardial necrosis/injury [22–24]. The largest disadvantage of hs-troponin measurements is that they often require serial meetings and are therefore time consuming and expensive [25]. Besides, diagnosing patients with low (serial) troponin measurement, but true UA remains a difficult challenge. There seems no benefit of early revascularisation or intensified antiplatelet therapy in terms of mortality in UA patients, but a considerable amount of UA patients do show obstructive CAD requiring planned revascularisation. They also show higher chances of future MI, indicating the importance of identifying these patients.

### Incidence of UA

The incidence of definite UA in our (complete) cohort was 5%. This is in line with recently published results by Puelacher et al., who investigated the incidence and outcomes of UA patients compared to NSTEMI patients in 8992 patients from the international APACE study and patients from a stepped wedge cluster RCT (4739 patients) which is still ongoing (HighSTEACS) [10]. However, wide ranges of incidence rates are reported, most likely as a consequence of an absent universal definition for UA [7]. Most guidelines use the absence of elevated troponin, or absence of rise and fall in combination with typical symptoms [1, 26, 27]. This is reflected in our cohort as well, since 13% of the patients were adjudicated as “possible UA”, meaning typical symptoms, absence of evidence for myocardial injury but non conclusive presence of ischemia.

### Plasma EV cystatin c

We found plasma EV protein cystatin c in the TEX subfraction (adjusted for confounders) to be associated with the presence of definite UA among patients with chest pain and low levels of hs-TnI. After determination of an optimal clinical cut-off and dichotomising of patients in either high or low this became even more pronounced. Cystatin c is an inhibitor of proteases that play a key role in inflammation. It is produced and secreted by cardiomyocytes and its synthesis is elevated when the myocardium experiences ischemia [28]. Plasma cystatin c is known as an important marker for renal dysfunction and also for its close relationship with CAD [29–31]. De Hoog et al. also showed that EV cystatin c was associated with ACS in the TEX subfraction in male patients [32]. Our Study has relatively low numbers, prohibiting to investigate whether the association of plasma EV cystatin c differed among sex. Considering the exploratory nature of this study, also no conclusions with regards to diagnostic properties of this marker can be made based on these results. Our findings should be considered as hypothesis generating only.

### Biomarkers and ischemic heart disease

Several new biomarkers have been proposed as marker for the early detection of ACS. Best known are; heart-type fatty acid-binding protein (hFABP), Copeptin, hs-CRP, Natriuretic peptides, ischemia modified albumin (IMA) and GDF-15 [25]. However, none of them are elevated as early as hs-TnI or as specific as troponin. miRNA’s have been studied extensively as well, but also lack sufficient evidence and are often very costly [33]. In contrast to end-products of cell stress (e.g. BNP, Copeptin) differences in EV protein levels represent differences upon cell-level. EV content might change already in a very early stage of the disease depicting more subtle but also more chronic conditions of disease. This difference might be the explanation for the great potential of EV proteins as diagnostic biomarker. EVs have shown to play an important role in the development and progression of atherosclerotic disease [34–37]. EV proteins have also been shown to be associated with cardiometabolic risk in the Framingham cohort [38] and are independent predictors for future cardiovascular events [16, 17]. In this study we have shown that there is a potential role for EV cystatin c in the detection of UA in patients with acute chest pain and low levels of troponin. Although our results are promising, future studies are needed to investigate whether the use of EV cystatin c could also improve clinical decision making and thereby improve patient care.

### Strengths and limitations

This study was a sub analysis from a prospective single centre study among acute chest pain patients. Although this was a single centre study, the Meander Medical Centre is known as a large hospital with high numbers of patients which makes the study results easily generalisable to the general ‘acute chest pain’ patient. A limitation of the study is the relatively small sample size, therefore the results of this study need to be interpreted with caution. A clear strength is the feasibility and clinical potential of the method used. The complete analysis could be performed within 2 hours in any clinical lab and only one droplet of blood is needed. The fractionation is relatively cheap and is suitable for automation. The readout is a standard readout immunobased assay (e.g. ELISA, MSD or Luminex). Thus, considering the use of existing, commonly used products and the possibility for automation of the isolation method we think the clinical implementation of our analysis method would be feasible. But first, larger studies are needed to confirm our findings and determine the diagnostic properties of this new potential biomarker.

### Conclusion

Despite the use of hsTn, UA remains important and patients require additional therapy. Therefore, additional risk stratification in patients with acute chest pain and low levels of hsTn is needed. In this exploratory, hypothesis generation study, we showed an association between low levels of EV plasma cystatin c and the presence of UA in patient with acute chest pain and low levels of hsTnI. Larger studies are needed to confirm our findings but EV plasma proteins should be considered as a promising tool for additional risk stratification in acute chest pain patients.

## Supporting information

S1 Appendix Supplemental methods regarding the isolation method. (DOCX)

S1 Fig Extracellular vesicle analysis procedure. Sequential isolation of plasma fractions and subsequential lysis and analysis of extracellular vesicles.
(DOCX)

S2 Fig Performance diagram to determine optimal cut-off values. Optimal cut-off decision curve for cystatin C and definite UA as determinant.
(DOCX)

S1 Table Heart score algorithm. (DOCX)

S2 Table Baseline extracellular vesicle protein levels. (DOCX)
