# Supplementary material for: Extracellular Vesicle cystatin c is associated with unstable angina in troponin negative patients with acute chest pain
Source: PLoS One. 2020 Aug 5;15(8):e0237036. doi: 10.1371/journal.pone.0237036 (PMC7406038; doi:10.1371/journal.pone.0237036)
Supplement: S1 Appendix — (DOCX) [file pone.0237036.s001.docx]

**S1 Appendix**

**Supplemental method**

Isolation of extracellular vesicle plasma subfractions

The isolation process of extracellular vesicles from plasma is shown in S1 Fig1. LDL and HDL subfractions can be obtained from plasma with Dextrane Sulphate (DS) and Manganese Chloride (MnCl2) in different solutions. For DS: 0.05%, MnCl2: 0.05M and DS: 0.65%, MnCl2: 0.2M, respectively. For LDL subfraction isolation, 25uL plasma was diluted in 80uL phosphate buffered saline (PBS) (Gibco), followed by addition of 5µL Nanomag®-D plain, 130mm (1:25) (Micromod). 15µL of a standard amount of synthetic liposomes, coated with DSG-PEG2000 (Nanocs) and fluorescently labeled with 18:1 liss rhod pe (Merck), was added to each plasma sample to be able to correct for loss of the pellet during isolation. DS and MnCl2 were added into the total volume of 125 μL and were mixed. The mixture was incubated 5 min at room temperature (RT). Subsequently, the samples were placed on a bio-plex handheld magnet (Bio-Rad) and incubated 15 min at RT. The pellets were lysed with 125µL Roche complete lysis-M with protease inhibitors (Roche). To remove magnetic beads and other debris, samples were centrifugated at 3200xg, 10 min. Fluorescence of the synthetic liposomes were measured with SpectraMax® Multi-Mode Microplate reader (Molecular Devices) directly after completion of the isolation protocol. For HDL isolation, the protocol is repeated when using 115µL supernatant above the LDL pellet. For the TEX subfraction, 25µL plasma was diluted in 80µL PBS, 5µL Nano-mag®-D PEG-OH (1:25) (Micromod) and 15µL of the synthetic fluorescent labelled liposomes. Xtractt buffer was added and the samples were mixed. The protocol is repeated identical to the LDL isolation procedure. The pellet is used as TEX subfraction.

Quantification of extracellular vesicle proteins

Concentrations of the selected proteins were determined using an electrochemiluminescence Immunoassay Instrument (Quickplex SQ120, Meso Scale Discovery, MSD). Specific designed 96-wells plates (MSD), incorporated with electrodes on the bottom, were used to measure the proteins in the subfractions. U-Plex Development Assay (MSD) is performed using manufacturers protocol.

The spots in the 96-wells plates were coated with capture antibodies for the protein of interest and were then stored in 4°C until use. Subsequently, the plates were blocked with blocking buffer (MSD) for 1 hour on a bench desk. Protein lysates from the EV subfractions were diluted in diluent buffer (MSD). Plates were then washed three times with 150uL of wash buffer (0.2% Tween-20 in PBS) per well. After washing the plates, the samples were added to the 96-wells plates and incubated overnight at 4°C. Next day, the 96-wells plates were washed as before and then incubated with detection antibody for 1 hour while shaking. After one hour, samples were washed again and Reading Buffer (MSD) was added. Samples were read within 10 min on a MSD Instrument. Protein concentration were measured as pg/mL. The data were analyzed using SoftMax Pro 7 Software (Molecular Devices).
