# Supplementary material for: Extracellular Vesicle cystatin c is associated with unstable angina in troponin negative patients with acute chest pain
Source: PLoS One. 2020 Aug 5;15(8):e0237036. doi: 10.1371/journal.pone.0237036 (PMC7406038; doi:10.1371/journal.pone.0237036)
Supplement: S1 Fig — Sequential isolation of plasma fractions and subsequential lysis and analysis of extracellular vesicles. (DOCX) [file pone.0237036.s002.docx]

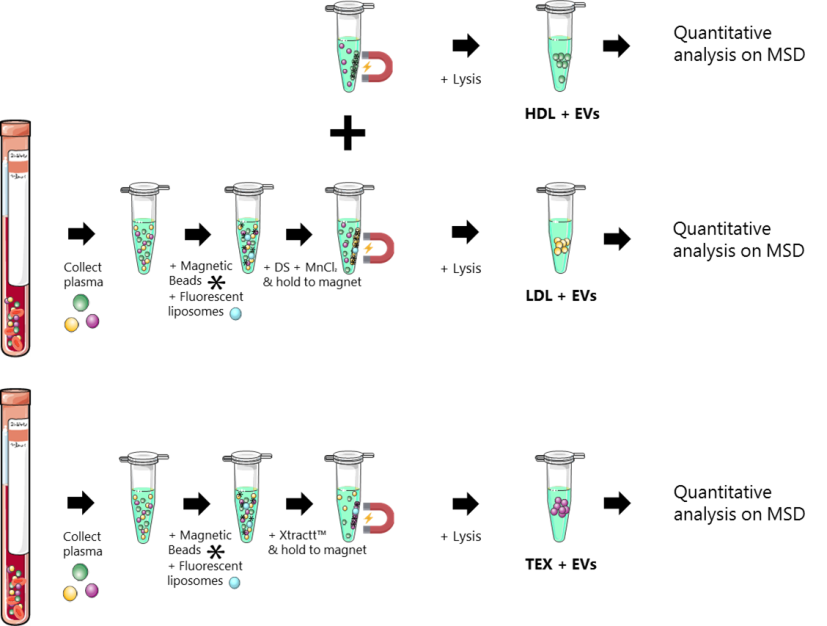


Supplemental figure 1. Extracellular vesicle analysis procedure
Sequential isolation of plasma fraction and subsequential lysis and analysis of extracellular vesicles.
