# Supplementary material for: Extracellular Vesicle cystatin c is associated with unstable angina in troponin negative patients with acute chest pain
Source: PLoS One. 2020 Aug 5;15(8):e0237036. doi: 10.1371/journal.pone.0237036 (PMC7406038; doi:10.1371/journal.pone.0237036)
Supplement: S2 Fig — Optimal cut-off decision curve for cystatin C and definite UA as determinant. (DOCX) [file pone.0237036.s003.docx]

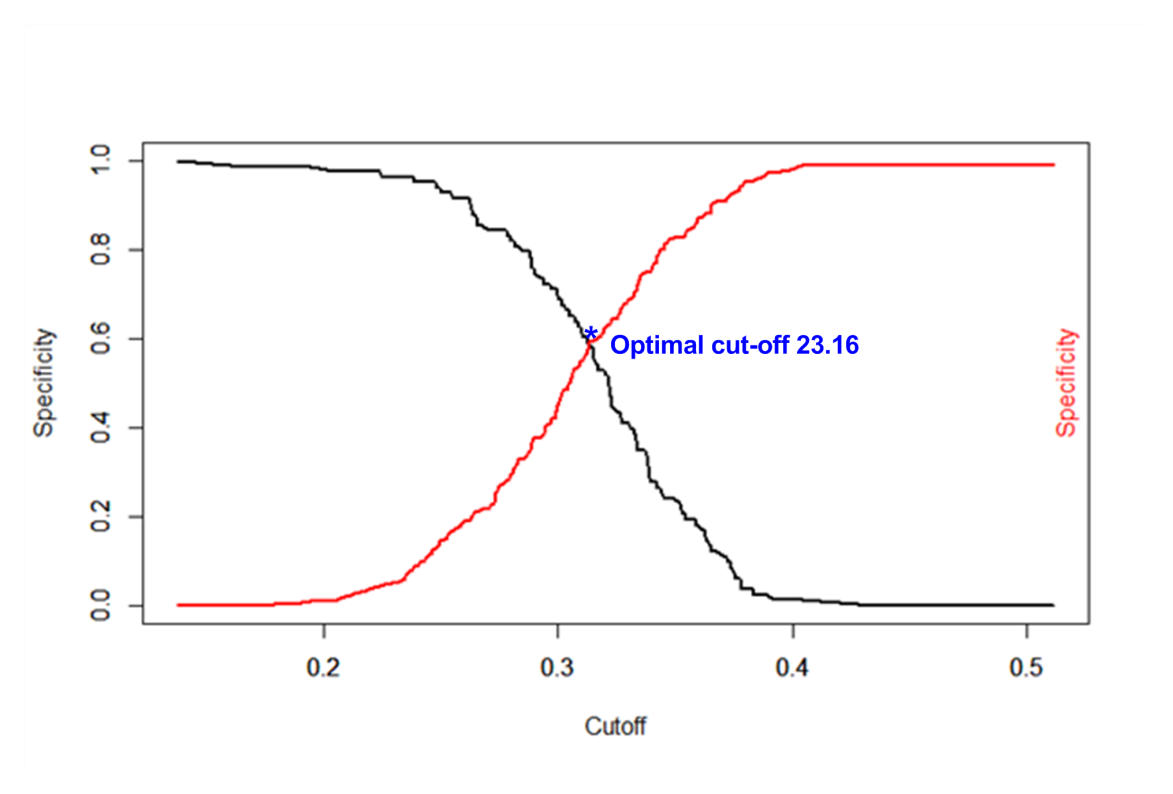


Supplemental figure 2. Performance diagram to determine optimal cut-off values
Optimal cut-off decision curve for cystatin C and definite UA as determinant.
