# Supplementary material for: Extracellular Vesicle cystatin c is associated with unstable angina in troponin negative patients with acute chest pain
Source: PLoS One. 2020 Aug 5;15(8):e0237036. doi: 10.1371/journal.pone.0237036 (PMC7406038; doi:10.1371/journal.pone.0237036)
Supplement: S1 Table — (DOCX) [file pone.0237036.s004.docx]

| **Supplemental table 1. HEART score algorithm** | | |
| --- | --- | --- |
| **Variable** | **Description** | **Score** |
| **H**istory | Highly syspicious | 2 |
|  | Moderately suspicious | 1 |
|  | Slightly/not suspicous | 0 |
| **E**CG | Significant ST depression | 2 |
|  | Nonspecific repolarization disturbances | 0 |
|  | Normal | 1 |
| **A**ge | ≥65 years of age | 2 |
|  | 45-65 years of age | 1 |
|  | ≤45 years of age | 0 |
| **R**isk factors | ≥3 Risk factorsᶧ, or history of CVD^1^ | 2 |
|  | 1 or 2 risk factors | 1 |
|  | No risk factors | 0 |
| **T**roponin^2^ | ≥3 times normal limit | 2 |
|  | 1-2 times normal limit | 1 |
|  | ≤ normal limit | 0 |
| *ᶧHypertension, Diabetes Mellitus, current smoking, hypercholesterolemia, family history of coronary artery disease and obesity (BMI > 30). ^1^CVD = cardiovascular disease: history of myocardial infarction, previous coronary revascularization, stroke or peripheral artery disease. ^2^Troponin levels were measured with the Access AccuTnI+3 Troponin I assay on the UniCel DxI Immunoassay System (Beckmann Coulter, Brea, CA). The cutoff for MI was set at >60 ng/L at the coefficient of variation <10%. The limit of detection was 10 ng/L, and the 99th percentile cut‐off point of 42 ng/L.* | | |
