# Supplementary material for: Extracellular Vesicle cystatin c is associated with unstable angina in troponin negative patients with acute chest pain
Source: PLoS One. 2020 Aug 5;15(8):e0237036. doi: 10.1371/journal.pone.0237036 (PMC7406038; doi:10.1371/journal.pone.0237036)
Supplement: S2 Table — (DOCX) [file pone.0237036.s005.docx]

| **Supplemental table 2. Baseline Extracellular Vesicle protein levels** | | | |
| --- | --- | --- | --- |
|  | **Non-UAP** | **UAP** | **P-value** |
| **Biomarker** | 186 | 83 |  |
| CD14 HDL | 4.27 (0.56) | 4.24 (0.68) | 0.788 |
| CD14 LDL | 5.53 (0.41) | 5.57 (0.44) | 0.467 |
| CD14 TEX | 16.11 (3.38) | 15.74 (3.64) | 0.420 |
| CC HDL | 3.76 (1.51) | 3.67 (1.63) | 0.653 |
| CC LDL | 6.22 (0.46) | 6.16 (0.44) | 0.310 |
| CC TEX | 23.98 (4.93) | 22.89 (4.70) | 0.088 |
| SC1 HDL | 5.91 (0.75) | 5.90 (0.90) | 0.935 |
| SC1 LDL | 129.08 (35.45) | 126.49 (40.07) | 0.595 |
| SC1 TEX | 4.50 (0.48) | 4.45 (0.36) | 0.380 |
| SF2 HDL | 8.90 (0.27) | 8.84 (0.25) | 0.100 |
| SF2 LDL | 9.21 (0.26) | 9.19 (0.23) | 0.605 |
| SF2TEX | 94.29 (14.68) | 91.35 (11.69) | 0.108 |
| SG1HDL | 3.39 (0.46) | 3.28 (0.58) | 0.114 |
| SG1LDL | 9.24 (2.31) | 9.17 (1.94) | 0.800 |
| SG1TEX | 7.76 (1.50) | 7.39 (1.13) | 0.049 |
| *EV protein levels are displayed as mean(sd). Proteins were transformed to achieve a normal distribution* *and standardized per synthetic vesicle. Log transformation: CD14HDL and LDL, CC HDL and LDL, SC1 HDL and TEX, SF2 HDL and LDL, SG1 HDL. Square root transformation: CD14TEX, CCTEX, SC1LDL, SF2TEX, SG1LDL.and TEX* | | | |
